# Prostate-Specific Antigen Screening According to Health Professional Counseling and Age in the United States

**DOI:** 10.1155/2022/8646314

**Published:** 2022-01-06

**Authors:** Ray M. Merrill, Seth A. Otto, Eliza B. Hammond

**Affiliations:** Department of Public Health, College of Life Sciences, Brigham Young University, Provo, UT 84602, USA

## Abstract

**Background:**

In 2018, the US Preventive Services Task Force recommended that PSA screening for prostate cancer involve men aged 55–69, based on a personal decision following consultation with a health professional. PSA screening in men aged 70 or older should only occur if symptoms exist. This study identifies the association between having a PSA test in the past two years and whether or not there was consultation with a health professional about the benefits and/or harms of PSA screening.

**Methods:**

Analyses were based on data involving men aged 40 years or older, who responded to PSA related questions in the 2018 BRFSS survey.

**Results:**

Approximately 32.0% (14.6% for ages 40–54, 41.7% for ages 55–69, and 49.8% for ages 70 years and older) of respondents had a PSA test in the past two years. Approximately 81.7% of these men had talked with a health professional about the benefits and/or harms of PSA screening, with 42.4% having discussed the benefits and harms, 54.6% having discussed the benefits only, and 3.0% having discussed the harms only. The odds of a PSA test in the past two years in men having talked with a health professional about the benefits and harms of the test versus no talk are 10.1 (95% CI 9.3–10.8), in men who talked with a health professional about the benefits only versus no talk are 10.8 (95% CI 10.0–11.6), and in men who talked with a health professional about the harms only versus no talk are 3.9 (95% CI 2.9–5.1).

**Conclusion:**

PSA screening is most common in men aged 70 or older, which is counter to the US Preventive Task Force recommendation. Most men having a PSA test have talked with a health professional about the test, but the talks tended to focus on just the benefits of screening and not both potential benefits and harms.

## 1. Introduction

Prostate cancer is the most common nonskin cancer among men in the United States. In 2021, it accounted for an estimated 25.6% (248,530) of all cancer cases in males and 10.7% (34,130) of all cancer deaths in males [1]. There are over 3.2 million men currently alive in this country with a previous diagnosis of the disease [2]. The average lifetime risk of prostate cancer is 11.7% (1 in 8.5) for whites and 16.7% (1 in 6) for blacks, based on 2016–2018 Surveillance, Epidemiology, and End Results (SEER) data [3]. Average lifetime risk of dying from prostate cancer is 2.3% (1 in 43.5) for whites and 3.8% (1 in 26.3) for blacks [3]. These results are consistent with prostate cancer being a slow-growing tumor such that men tend to die with the disease rather than from it [4–6].

Screening, diagnosis, and treatment for prostate cancer has been controversial in the past because of over diagnosis [7, 8]. Prostate cancer screening may be associated with a decrease in prostate cancer death, though the evidence for this is conflicted [9]. Potential harmful effects of PSA screening include false-positive results and complications associated with resulting biopsy and treatment [9], such as adverse urinary symptoms and sexual dysfunction [10, 11]. Nevertheless, in the past decade, important advances allow us to better characterize the likely progression of the disease following diagnosis and more effective treatment options are now available to patients [7, 12–16].

Previous reviews have summarized screening, diagnosis, and treatment options for prostate cancer [17–19]. Measurement of prostate-specific antigen (PSA) protein in the blood is the most common screening approach used for prostate cancer. In 2018, the US Preventive Services Task Force provided a recommendation statement regarding prostate cancer screening [20]. For men aged 55 to 69 years, PSA-based screening for prostate cancer should be an individual decision. The decision should follow a talk with their doctor about the clinical benefits (identifying high-risk early stage prostate cancer, which can be successfully treated) and harms (false positives, overdiagnosis, and treatment complications) of screening for prostate cancer. For men 70 years and older, routine PSA-based screening for prostate cancer is not recommended in the absence of symptoms. These recommendations apply to men of average or increased risk for prostate cancer who do not have symptoms of the disease and who have not been already diagnosed with prostate cancer.

The purpose of this study was to identify the prevalence of PSA testing in men aged 40 years or older, reasons for the test, and whether the respondents had ever talked with a health professional about the benefits and/or harms of PSA screening. The study assessed the association between having a PSA test in the past two years according to whether or not a health professional had talked with them about the benefits and/or harms of PSA screening or had recommended the test.

## 2. Methods

### 2.1. Data

The Behavioral Risk Factor Surveillance System (BRFSS) is a project wherein states in the United States (US) and participating US territories collaborate with the Center for Disease Control and Prevention (CDC). The BRFSS involves ongoing health-related telephone surveys designed to collect information on health-related risk behaviors, chronic health conditions, and access to preventive services. It is administered to noninstitutionalized adults in the US, aged 18 years and older [21]. The survey utilizes a random digit dialing technique on both cell phones and landlines to gather participants. Mean percent response rates for the 2018 BRFSS participating areas are 53.4% for landlines and 46.0% for cell phones (49.8% combined) [22].

Analyses are restricted to 129,989 men aged 40 years or older who responded to whether or not they previously had a PSA test. Of this number, 3,806 had the test to monitor prostate cancer. These respondents were eliminated in our evaluation of PSA as a screening test, resulting in 126,183 respondents for evaluation.

### 2.2. Variables

Outcome variables include prevalence of a PSA test, reasons for the PSA test, whether or not a health professional had previously talked with the survey respondent about the benefits and/or harms of PSA screening, whether PSA screening had been recommended, and the percent of men who had a PSA test in the past two years. These outcome variables were determined from the following questions: “Have you ever had a PSA test?” “How long has it been since you had your PSA test?” “What are the main reasons you had this PSA test?” “Has a doctor, nurse, or other health professional ever talked with you about the benefits of the PSA test?” “Has a doctor, nurse, or other health professional ever talked with you about the harms of the PSA test?” “Has a doctor, nurse, or other health professional ever recommended that you have a PSA test?” These questions are listed in the 2018 BRFSS Codebook Report (https://www.cdc.gov/brfss/annual_data/2018/pdf/codebook18_llcp-v2-508.pdf) [23].

Outcome variables were associated with and adjusted for race/ethnicity, age, marital status, education level, annual household income, BMI weight classification, whether they had smoked 100 or more cigarettes in the past, family history of prostate cancer, and health insurance. Race/ethnicity was classified as non-Hispanic white, non-Hispanic black, non-Hispanic Asian, non-Hispanic American Indian/Alaskan Native, Hispanic, and non-Hispanic other race. Hereafter, we refer to these groups as white, black, Asian, American Indian/Alaskan Native, Hispanic, and other races. Questions upon which these variables are based are also included in the 2018 BRFSS Codebook Report [23].

### 2.3. Statistical Techniques

Frequencies, percentages, and odds ratios were calculated and reported from the sample survey data. Multiple logistic regression was used to estimate odds ratios adjusted for potential confounders. Odds ratios measured the association between having had PSA screening in the past two years and ever having had a talk with a health professional about the benefits and/or harms of PSA screening. Other variables were also associated with PSA screening in the past two years: race/ethnicity, age, marital status, education level, annual household income, smoking status, family history of prostate cancer, and health insurance. Person-level weights were applied to generate population estimates. Weighted percentages and odds ratios were reported in this paper. Statistical analyses were performed using SAS 9.4 (SAS Institute, Cary, NC, USA, 2012). SAS procedures used in the current study were SURVEYFREQ and SURVEYLOGISTIC. Graphs were created in Microsoft Excel (Microsoft Corporation, Issaquah, WA, USA, 2016).

## 3. Results

Selected aspects of PSA testing for men aged 40 years or older according to race/ethnicity appear in Table 1. Approximately 45.3% of men have ever had a PSA test, with the prevalence highest in whites and blacks and lowest in Asians and Hispanics. Having a PSA test as part of a routine exam is the most likely reason for the test, more so among Asians and Hispanics. Having a PSA test because of a prostate problem is also more common in Asians and Hispanics. Having a PSA test because of a family history of prostate cancer is more common in American Indians/Alaskan Natives. Finally, having a PSA test because of prostate cancer is more common in whites and blacks. Having ever talked with a health professional about the benefits of PSA screening, talked about the harms of PSA screening, been recommended PSA screening, or undergone a PSA screen in the past two years are each significantly more likely in whites and blacks. Talking about the benefits is approximately twice as common as talking about the harms, similarly across the racial/ethnic groups.

**Figure 1:**
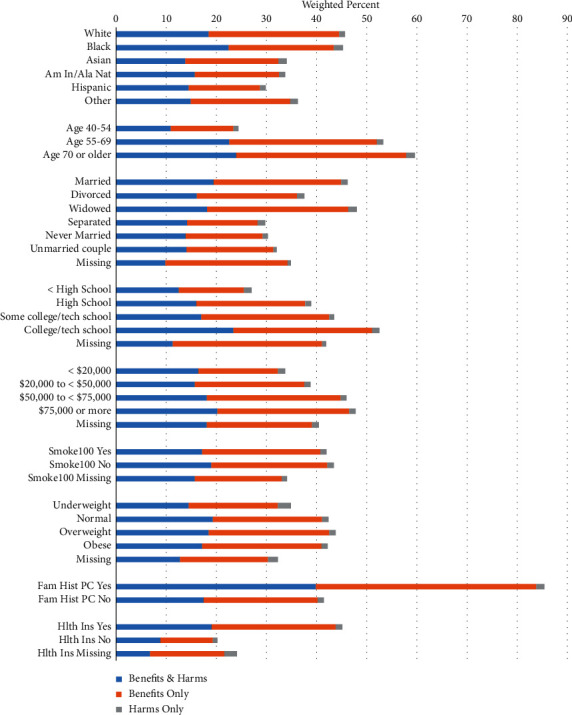
Health professional ever talked with the survey respondent about benefits and/or harms of PSA screening according to selected variables.

The percent of respondents who indicated that a health professional had ever talked with them about benefits and/or harms of PSA screening is shown according to the level of selected variables in Figure 1. A pattern of greater talk about only the benefits of PSA screening appears across the levels of each of the variables in the figure. In general, ever having talked about PSA screening with a health professional increased with age (24.5% in ages 40–54, 53.3% in ages 55–69, and 59.6% in ages 70 or older). Ever having talked with a health professional about both the benefits and harms of PSA screening occurred in 18.1% (10.9% in ages 40–54, 22.6% in ages 55–69, and 24.0% in ages 70 or older) of men, about the benefits only in 23.3% (12.5% in ages 40–54, 29.3% in ages 55–69, and 33.8% in ages 70 or older) of men, and about the harms only in 1.3% (1.0% in ages 40–54, 1.3% in ages 55–69, and 1.8% in ages 70 or older) of men. Thus, it is most common for a health professional to talk about the benefits only, followed by both the benefits and harms and then the harms only. The ratio of talk about just benefits versus talk about both benefits and harms increased across the three age groups, from 14.7% greater to 29.6% greater and to 40.8% greater.

**Figure 2:**
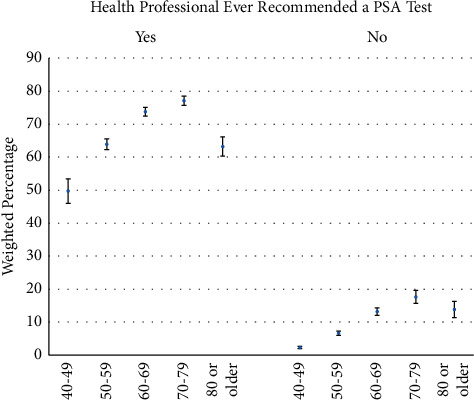
PSA test in the past two years according to a previous recommendation for the test from a health professional and age.

The percent of men who had a PSA test in the past two years according to whether they had a previous recommendation for the test from a health professional is shown in Figure 2. In each age group, the percent having a PSA test in the past two years was strongly influenced by whether it was recommended by a health professional. Specifically, if PSA screening was ever recommended versus otherwise, having a PSA test in the past two years was 21.5 times greater in ages 40–49, 9.6 times greater in ages 50–59, 5.6 times greater in ages 60–69, 4.4 times greater in ages 70–79, and 4.6 times greater in ages 80 or older. The percent of respondents ever recommended PSA screening was 13.8% in ages 40–49, 38.2% in ages 50–59, 55.2% in ages 60–69, 62.0% in ages 70–79, and 52.6% in ages 80 or older. Overall, when a doctor, nurse, or other health professionals ever recommended PSA screening, 68.8% had a PSA test in the past two years, compared with 7.5% otherwise.

PSA screening in the past two years was significantly greater among men who had a health professional ever talk with them about the benefits and/or harms of PSA screening (Table 2). Although talking about the benefits is most strongly associated with PSA screening in the past two years, talking about the harms only was also positively associated with PSA screening in the past two years. Without adjustment for potential confounders, whites were significantly more likely to receive PSA screening in the past two years. However, after adjusting for education, annual household income, and other variables, blacks and Hispanics (versus whites) were significantly more likely to have PSA screening in the past two years. PSA screening in the past two years was also positively associated with age and was more common among men who were married, had higher education, had higher annual household income, had not smoked at least 100 cigarettes in their lifetime, were overweight or obese, had a family history of prostate cancer, and were currently insured.

Prevalence of having had a PSA test in the past two years among men aged 40 years and older is 32.0% (14.6% for ages 40–54, 41.7% for ages 55–69, and 49.8% for ages 70 years and older). Prevalence of ever having discussed the benefits and/or harms of a PSA test with a health professional for these men who received the test was 81.7% (76.6% for ages 40–54, 83.3% for ages 55–69, and 82.3% for ages 70 years and older).

**Figure 3:**
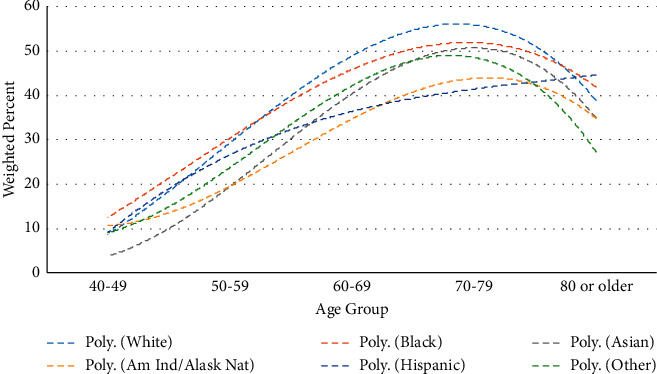
PSA test in the past two years according to age and race/ethnicity (data fit using third-order polynomial models).

PSA screening in the past two years according to age and race/ethnicity appears in Figure 3. PSA screening is most common in the age group 70–79 years, except for Hispanics, where it is the greatest in the age group 80 or older. In the age groups 40–49 and 50–59, PSA screening in the past two years is most common among blacks and least common among Asians. In the age groups 60–69 and 70–79, PSA screening in the past two years is most common among whites and blacks and least common among American Indians/Alaskan Natives and Hispanics. In the age group 80 years or older, PSA screening in the past two years decreases, except for among Hispanics.

## 4. Discussion

This study identified the prevalence of a health professional ever having talked with the survey respondents about the benefits and/or harms of PSA screening, according to age and other variables. It also investigated how this talk and recommendation for PSA screening is associated with PSA screening in the past two years. Further, we presented the level PSA screening in the past two years according to age and other variables.

Previous research indicates that PSA screening has the potential of overdiagnosing patients and increasing prostate cancer incidence among men more likely to die from other causes before prostate cancer symptoms manifest themselves [4–8, 24]. Potential harmful effects of PSA screening (e.g., false-positive results and complications associated with resulting biopsy and treatment [9–11, 17, 25]) led the US Preventive Services Task Force to recommend that for men aged 55 to 69, PSA-based screening for prostate cancer should be an individual decision, following a talk with their doctor about the benefits and harms of screening for prostate cancer [20]. For men 70 years and older, routine PSA-based screening for prostate cancer should not be done for men who do not have symptoms of the disease [20].

It is recommended that men have the chance to discuss the benefits and harms of PSA screening with their primary care provider as they make decisions about screening [25, 26]. Nevertheless, only 42.7% (24.4% in ages 40–54, 53.2% in ages 55–69, and 59.6% in ages 70 or older) of the men surveyed reported having previously had a talk with a health professional about the benefits and/or harms of PSA screening. Further, the talks tended to focus on just the benefits of PSA screening (i.e., 23.3%, 12.5% in ages 40–54, 29.3% in ages 55–69, and 33.8% in ages 70 or older) and not on both the benefits and harms of PSA screening (i.e., 18.1%, 10.9% in ages 40–54, 22.6% in ages 55–69, and 24.0% in ages 70 or older). Talking about just the harms of PSA screening occurred less than 2% of the time. Thus, in most of the cases where a health professional talked with the participants about PSA screening, the talk was not a balanced discussion and the ability of the man to make an informed decision was comprised.

PSA screening in the past two years peaked in ages 70–79 and decreased thereafter. A drop in the oldest ages may be motivated by recommendations discouraging PSA screening in older ages [20, 25]. These recommendations are influenced by prostate cancer being a slow growing tumor and five-year survival improving with older age [4–7, 27, 28]. Nevertheless, the high level of PSA screening occurring in the ages 70–79 and 80 or older is in contrast to what is recommended by the US Preventive Services Task Force.

Recommendation for PSA screening significantly positively associated with PSA screening in the past two years. The impact of recommendation on PSA screening was most pronounced in the younger age groups. Recommendation for PSA screening in a previous study showed that PSA testing is positively associated with a physician's direct communication about prostate cancer and encouragement to be screened [26].

In the remainder of this discussion, we will talk about the results involving race/ethnicity and other selected variables. Whites and blacks were more likely to have ever had a talk with a health professional about the benefits and/or harms of PSA screening. Whites and blacks were also more likely to have a health professional ever recommend PSA screening. These results may be because blacks have significantly higher rates of prostate cancer than the other racial/ethnic groups, followed by whites [29]. In addition, whites and blacks have the highest level of a family history of prostate cancer and whites and Asians have the highest levels of marriage, education, income, and health insurance (data not shown), each of which has been associated with increased levels of prostate cancer screening [30–32].

The significantly higher level of PSA screening in the past two years among whites and blacks is consistent with their having a higher level of health professionals talk with them about the benefits and/or harms of PSA testing, as well as recommending the test. However, blacks and Hispanics had the lowest levels of education, income, and insurance among the racial/ethnic groups (data not shown), which are buoying factors for higher PSA screening.

In the adjusted model (Table 2), blacks and Hispanics, compared with whites, experienced higher PSA screening in the past two years.

PSA screening in the past two years shows an increase until peaking in the age group 70–79 and then decreases. This is true for all racial/ethnic groups except for Hispanics, where the rate is greatest in the age group 80 or older. According to the guidelines [20], the decrease in PSA screening should have peaked at least a decade earlier for each of the racial/ethnic groups. However, the rate of increase with increasing age groups is decreasing for each racial/ethnic group through ages 70–79.

In the adjusted model (Table 2), married men had significantly higher levels of PSA testing in the past two years. This is consistent with previous literature showing that men who have a spouse or partner are more likely to be screened for prostate cancer [33–35]. This is likely because they have an independent party involved and invested in their health.

Higher education and higher annual household income were both associated with significantly greater PSA screening in the past two years. These two variables are usually connected to the broader term—socioeconomic status—a key determinant of health [36]. As such, higher education and income have been closely linked to greater PSA screening [37, 38]. Higher education and income are associated with increased knowledge and access to quality healthcare, which allows a patient to better understand and react to their situation. Thus, not only are these patients likely to be more informed about prostate cancer and PSA screening overall, but also they are better equipped to obtain the PSA test because of their greater financial resources.

Men who smoked at least 100 cigarettes in their lifetime were significantly less likely to have had a PSA test in the past two years. Other research has shown that current smokers are significantly less likely to undergo PSA screening [39, 40]. In the current study, lower PSA screening among smokers persisted after adjusting for several other variables. Smoking may represent a host of poor health behaviors, such as compromised consumption of healthy food, inadequate exercise, and risky sexual behavior [41, 42].

The analysis showed that overweight and obese individuals were more likely to have had a PSA test in the past two years. These results became more pronounced in the adjusted model. Previous research has likewise shown that overweight and obesity are associated with greater PSA testing [43]. This may be due to existing health concerns or comorbid factors that cause these individuals to be more conscientious of arising health issues. Additionally, it is possible that these individuals, because they are less healthy, are more likely to frequently check-in with their doctor [44].

Individuals with a family history of prostate cancer were significantly more likely to have a PSA test in the past two years. This group was far more likely than any other group (i.e., 85.4%) to have ever had a talk with a health professional about the benefits and/or harms of PSA screening. Approximately 39.9% had ever had a talk with a health professional about the benefits and harms, 43.9% had ever had a talk with a health professional about the benefits only, and 1.6% had ever had a talk with a health professional about the harms only. Among the variables considered in this study, ever having talked with a health professional about the benefits and/or harms of PSA screening and a family history of prostate cancer were the two leading factors associated with a PSA test in the past two years.

Those with some form of health insurance (prepaid plans or government plans) were also more likely to undergo PSA screening in the past two years. Other research has shown a direct association between health insurance and cancer testing, specifically involving cervical cancer, colon cancer, and mammography [45].

Some limitations exist in this study. First, response rates average 49.8%. Low response rates may have resulted in selection bias. Research has shown that telephone-based survey response rates have recently decreased and are lower than in-person response rates. However, similar research findings have indicated that applying weight to demographic variables of participants generally allows for accurate measurements [21]. Second, this is a cross-sectional survey where poor recall may be an issue. Third, interpretation is limited to discussing associations rather than cause-effect relationships.

## 5. Conclusion

### 5.1. PSA Screening

Although the US Preventive Services Task Force general guidelines recommend that men begin PSA screening at age 55 and stop at age 70, unless symptoms are present, a large proportion of men in the age range 40–54 and 70 and older undergo PSA screening. In our US sample, PSA screening in the past two years is greatest among men ages 70–79 years. The current study shows that talking with a health professional about PSA screening or receiving a recommendation for screening has a large impact on the decision to be screened. Hence, health professionals should be more sensitive to the age guidelines for screening as they counsel patients.

### 5.2. Informed Decision-Making

Undergoing PSA screening for prostate cancer should be an individual decision, but in conjunction with consultation with a health professional about the potential benefits and harms of screening. In our sample of men who were PSA screened in the past two years, approximately 81.7% (76.6% in ages 40–54, 83.3% for ages 55–69, and 82.3% for ages 70 years and older) had previously talked with a health professional about the benefits and/or harms of PSA screening. Hence, there is a need for many men undergoing PSA testing who have not first talked with a health professional about both the potential benefits and harms of the test to do so.

When talks occurred with a health professional about PSA screening, 42.4% involved discussion about the benefits and harms, 54.6% involved discussion about the benefits only, and 3.0% involved discussion about the harms only. Thus, health professionals should do a better job providing a balanced discussion with their patients of the benefits and harms of PSA screening.

### 5.3. Factors Associated with PSA Screening and Consultation

Other factors associated with higher levels of PSA screening are being white, being older in age, being married, being a college or technical school graduate, having an annual household income of $75,000 or more, not having smoked at least 100 cigarettes in their lifetime, being overweight or obese, having a family history of prostate cancer, and having health insurance. Having a health professional talk with a patient about the potential benefits and/or harms of PSA screening or having a family history of prostate cancer had the greatest positive association with PSA screening.

Health professionals are most likely to discuss benefits and/or harms of PSA screening with whites and blacks and least likely with Hispanics. They are also more likely to recommend PSA screening for whites and blacks. These differences in discussion and recommendation are likely explained, at least in part, by differences among the racial/ethnic groups in levels of marriage, education, annual household income, and insurance.

## Figures and Tables

**Table 1:** Prevalence of PSA testing, reasons for having the test, and talks with healthcare professionals about the test according to race/ethnicity.

	No.	%^1^	White %^1^	Black %^1^	Asian %^1^	Am. Indian %^1^	Hispanic %^1^	Other %^1^	Rao–Scott Chi-square *p*-value
Ever had a PSA test									
Yes	67620	45.3	49.0	44.8	30.8	34.3	33.3	38.4	<0.0001
No	62369	54.7	51.0	55.2	69.2	65.7	66.7	61.6	
Missing	7630								

PSA test in the past two years									
Yes	45067	32.0	34.7	30.7	22.6	24.1	23.7	26.4	<0.0001
No	79203	68.0	65.3	69.3	77.4	75.9	76.3	73.6	
Missing	1877								

Reason for having a PSA test									
Part of routine exam	47165	71.4	71.6	67.7	75.3	70.4	73.8	69.3	0.0005
Prostate problem	5174	7.6	7.2	8.0	9.0	6.9	9.1	7.5	
Family history of p.c.	3869	5.8	6.2	5.7	4.4	7.3	4.2	4.2	
Prostate cancer	3806	4.8	5.0	5.5	2.4	3.7	3.1	5.0	
Other reasons	6725	10.4	10.1	13.1	9.0	11.7	9.8	14.1	
Missing	881								

Doctor, nurse, or other health professional ever talked with you about the benefits of the PSA test^2^									
Yes	60594	43.7	47.10	45.76	34.19	34.31	29.81	37.52	<0.0001
No	62498	56.3	52.90	54.24	65.81	65.70	70.19	62.48	
Missing	3091								

Doctor, nurse, or other health professional ever talked with you about the harms of the PSA screennig^2^									
Yes	27854	20.0	20.4	25.2	16.0	17.8	16.1	17.2	<0.0001
No	94709	78.0	79.6	74.8	84.0	82.2	83.9	82.8	
Missing	3620								

Doctor, nurse, or other health professionals ever recommend that you have a PSA screening^2^									
Yes	56256	40.4	43.4	41.5	29.6	29.7	29.4	33.6	<0.0001
No	67050	59.6	56.6	58.5	70.4	70.3	70.6	66.4	
Missing	2677								

The results in this table apply to male respondents aged 40 years or older. ^1^Percentages are weighted in order to get a representative sample of the US population. ^2^Those who had a PSA test to monitor prostate cancer are not included.

**Table 2:** Odds of receiving a PSA test in the past two years according to whether or not a health professional had ever talked with the respondent about the benefits and/or harms of PSA testing.

				PSA in the past two years unadjusted				PSA in the past two years adjusted for the other variables in the table			
	No.	Col%	Row %	Odds ratio	95% LCL	95% UCL	Pr > |t|	Odds ratio	95% LCL	95% UCL	Pr > |t|
Talk about PSA with health professional											
Benefits & harms	27408	18.4	61.2	13.93	12.98	14.95	<0.0001	10.07	9.34	10.85	<0.0001
Benefits only	34532	23.9	63.3	15.24	14.20	16.35		10.76	9.99	11.58	
Harms only	1598	1.3	36.0	4.98	3.91	6.35		3.87	2.94	5.10	
Neither	66451	56.4	10.2	1				1			

Race/ethnicity											
White	100435	68.6	34.7	1			<0.0001	1			<0.0001
Black	8746	10.4	30.7	0.83	0.77	0.91		1.14	1.02	1.27	
Asian	2520	4.4	22.6	0.55	0.44	0.68		0.68	0.54	0.85	
Am. Indian/Alaskan Nat	2359	1.0	24.1	0.60	0.49	0.73		0.89	0.70	1.14	
Hispanic	8242	13.9	23.7	0.58	0.52	0.65		1.26	1.10	1.45	
Other	3881	1.8	26.4	0.68	0.59	0.78		0.97	0.81	1.16	

Age groups											
40–54	37152	41.3	14.6	1			<0.0001	1			<0.0001
55–69	54197	38.8	41.7	4.19	3.92	4.47		2.86	2.65	3.09	
70 or older	34834	20.0	49.8	5.80	5.39	6.25		3.84	3.51	4.21	

Marital status											
Married	78406	65.0	35.5	1			<0.0001	1			<0.0001
Divorced	19436	13.8	27.0	0.67	0.62	0.72		0.87	0.80	0.96	
Widowed	9739	5.5	37.1	1.07	0.96	1.19		0.81	0.72	0.93	
Separated	2685	2.5	20.0	0.45	0.38	0.55		0.79	0.64	0.97	
Never married	12751	10.3	19.6	0.44	0.41	0.49		0.73	0.65	0.82	
Unmarried couple	2720	2.6	21.8	0.51	0.42	0.60		0.79	0.64	0.98	
Missing	446	0.3	24.8	0.60	0.42	0.86		0.80	0.52	1.22	

Education level											
< High school	9678	14.2	19.9	0.61	0.55	0.68	<0.0001	0.82	0.72	0.94	<0.0001
High school	33847	27.1	29.0	1				1			
Some college/tech school	31726	28.5	32.9	1.20	1.12	1.29		1.04	0.96	1.13	
College/tech school	50686	30.0	39.7	1.61	1.52	1.71		1.18	1.09	1.27	
Missing	246	0.2	28.5	0.98	0.51	1.86		0.71	0.40	1.25	

Annual household income											
< $20,000	15098	12.5	22.7	0.72	0.65	0.79	<0.0001	0.88	0.79	0.99	<0.0001
$20,000 to <$50,000	33607	25.6	29.0	1				1			
$50,000 to <$75,000	18509	13.8	34.5	1.29	1.19	1.40		1.10	0.99	1.22	
$75,000 or more	44506	36.7	35.9	1.37	1.29	1.46		1.21	1.10	1.32	
Missing	14463	11.5	33.4	1.23	1.12	1.35		1.27	1.13	1.42	

Have smoked at least 100 cigarettes in lifetime											
Yes	64850	50.9	31.4	1			0.0430	1			<0.0001
No	60646	48.5	32.7	1.06	1.01	1.12		1.08	1.02	1.15	
Missing	687	0.6	29.9	0.931	0.70	1.24		1.26	0.88	1.80	

Body mass index category											
Underweight	954	0.7	23.7	0.70	0.55	0.90	<0.0001	0.89	0.67	1.17	<0.0001
Normal weight	27283	21.0	30.6	1				1			
Overweight	53374	42.4	33.1	1.12	1.05	1.20		1.14	1.05	1.24	
Obese	42285	33.7	32.5	1.09	1.02	1.17		1.25	1.14	1.36	
Missing	2287	2.2	19.8	0.56	0.47	0.68		0.77	0.59	1.01	

Family history of prostate cancer											
Yes	3869	2.7	74.0	6.39	5.46	7.48	<0.0001	3.37	2.67	4.24	<0.0001
No	122314	97.3	26.0	1				1			

Current health insurance status											
Yes	116996	90.0	34.2	3.89	3.34	4.54	<0.0001	1.74	1.47	2.05	<0.0001
No	8864	9.7	11.8	1.00				1			
Missing	323	0.3	22.2	2.13	1.25	3.64		2.01	1.11	3.63	

LCL: lower confidence level; UCL: upper confidence level. The results in this table apply to male respondents aged 40 years or older. Those who had a PSA test to monitor prostate cancer are not included. Four categories of BMI: underweight BMI <18.5, normal weight 18.5 ≤ BMI <25, overweight 25 ≤ BMI <30, and obese 30 ≤ BMI. Percentages and odds ratios (with corresponding confidence intervals) are weighted in order to get a representative sample of the US population. Confidence intervals that do not overlap 1 indicate statistical significance at the 0.05 level.

## Data Availability

The data used to support the findings of this study are available from the corresponding author upon request.

## Abbreviations

BRFSS: Behavior Risk Factor Surveillance System
CDC: Centers for Disease Control and Prevention
PSA: Prostates Specific Antigen
SAS: Statistical Analysis System
SEER: Surveillance, Epidemiology, and End Results
US: United States.
